# Tumor-immune spatiotemporal co-evolution as a paradigm for overcoming therapy resistance in advanced prostate cancer

**DOI:** 10.3389/fimmu.2026.1797299

**Published:** 2026-03-11

**Authors:** Junchao Xue, Kaisen Liao, Meng Zhang

**Affiliations:** Tongde Hospital of Zhejiang Province, Hangzhou, China

**Keywords:** cancer-associated fibroblasts, clonal-stromal co-selection, immune checkpoint blockade, prostate cancer immunotherapy, radiopharmaceutical therapy, spatiotemporal co-evolution, therapy resistance, tumor microenvironment

## Abstract

Therapeutic resistance in metastatic castration-resistant prostate cancer (mCRPC) is orchestrated not only by tumor-intrinsic genomic alterations but also by dynamic reprogramming of the tumor microenvironment (TME). This review introduces the tumor-immune spatiotemporal co-evolution paradigm, which reframes mCRPC resistance as an ecosystem-level adaptation unfolding across temporal (disease stage) and spatial (niche architecture) dimensions. We synthesize clinical and multi-omics data to map a probabilistic evolutionary trajectory from an immune-permissive state, through suppressive niche consolidation, to a terminal immune desert phenotype. In this review, we systematically apply the Oxford Centre for Evidence-Based Medicine (OCEBM) 2011 criteria to this field, grading all mechanistic claims to explicitly distinguish peer-reviewed, validated findings (Level 1–2b) from speculative hypotheses (Level 3–4), and delineate 5 evidence-graded core conclusions of the tumor-immune co-evolution paradigm. We delineate how spatially organized cancer-associated fibroblast (CAF) subsets architect immunosuppressive niches and engage in reciprocal metabolic symbiosis with tumor cells, and redefine therapeutics as dominant selective pressures that drive clonal-stromal co-selection to explain cross-resistance across treatment modalities. To translate this paradigm, we propose an integrative closed-loop *“Dynamic Monitoring—Mechanistic Parsing—Synergistic Intervention”* framework, with concrete, clinically actionable strategies grounded in 2024–2025 peer-reviewed prostate cancer research. This framework advocates for longitudinal ecological auditing of the TME to rationally guide mechanistically orthogonal combination therapies. Our objective is to provide a rigorously evidence-based roadmap for transforming mCRPC into a chronically manageable condition through precision ecological intervention, offering a novel, actionable perspective to advance prostate cancer immunotherapy and overcome immune evasion for researchers and clinicians in the field of cancer immunology.

## Highlights

- The prostate tumor–immune ecosystem undergoes predictable spatiotemporal co-evolution across disease stages, with paired patient specimen analyses confirming progressive immune suppression from hormone-sensitive prostate cancer (HSPC) to mCRPC (Level 2b).
- CAFs drive immunosuppression through two core mechanisms: (1) subset heterogeneity, supported by correlative evidence from human mCRPC spatial transcriptomics (Level 3); (2) reciprocal metabolic symbiosis with tumor cells, supported exclusively by preclinical functional validation (Level 4, unvalidated hypothesis).
- Standard-of-care therapeutics act as dominant selective pressures that remodel the tumor-immune ecosystem via clonal–stromal co-selection, providing a mechanistic explanation for cross-resistance across androgen receptor pathway inhibitors (ARPIs), chemotherapy, and radiopharmaceutical therapy (RPT) (Level 2b).
- The closed-loop *“Dynamic Monitoring—Mechanistic Parsing—Synergistic Intervention”* framework enables precision ecological management of mCRPC, with concrete biomarker and trial design strategies informed by completed phase II clinical trials (Level 2b).
- Prospective longitudinal trials with embedded ecological auditing are required to validate this paradigm and translate it into biomarker-stratified combination therapies (Level 3 hypothesis).

## 1 Introduction: the imperative for an ecosystem-centric perspective

The transition from localized prostate cancer (PCa) to lethal mCRPC and therapy-induced neuroendocrine prostate cancer (NEPC) constitutes a canonical exemplar of therapeutic adaptation and lineage plasticity (1). For decades, the oncologic community has approached this progression through a cell-autonomous lens, attributing resistance to tumor-intrinsic genomic alterations: *AR* amplifications, *TP53/RB1* biallelic loss, and *BRN2*-mediated neuroendocrine reprogramming (1, 2). While these mechanisms provide a partial molecular scaffold, they fail to explain pervasive intra-patient heterogeneity, inevitable relapse across all targeted therapies, profound cross-resistance between nominally distinct treatment modalities, and the consistent lack of durable clinical benefit from immune checkpoint blockade (ICB) in unselected patient populations—an unmet clinical need that remains a central challenge in PCa immunology and immunotherapy (3, 4).

Paralleling this genetic narrative, the constitutively immunologically “cold” prostate TME has emerged as a dominant non-cell-autonomous determinant of therapeutic failure across endocrine therapy, taxane chemotherapy, radiopharmaceuticals, and ICB (5, 6). Unlike immunogenic solid tumors such as melanoma or non-small cell lung cancer, PCa is characterized by a low tumor mutational burden and a baseline immunosuppressive TME, making the spatiotemporal co-evolution of tumor and stromal compartments the dominant driver of therapeutic resistance, rather than neoantigen-driven immunoediting alone. We posit that these ostensibly parallel narratives are causally linked through tumor-immune spatiotemporal co-evolution: dynamic, reciprocal crosstalk between malignant clones and their protective ecosystem that unfolds across both temporal (disease progression stages) and spatial (intra-tumoral niche architecture) dimensions. This framework extends seminal principles of cancer immunoediting (7) and the cancer-immunity cycle (8), with a critical novel emphasis: therapeutic pressure acts as the dominant selective force that actively sculpts ecosystem composition and function through clonal-stromal co-selection.

The central thesis of this review is that reframing mCRPC resistance as an ecosystem-level adaptive process—rather than solely a tumor cell-intrinsic phenomenon—provides a transformative lens for therapy design. This perspective necessitates three core shifts in clinical and translational practice: longitudinal ecological auditing of the TME, mechanistically-informed therapeutic combinations targeting integrated ecosystem states, and adaptive treatment sequencing guided by real-time biomarker assessment. We systematically synthesize multi-dimensional evidence for this paradigm, rigorously distinguishing empirically validated mechanisms from testable hypotheses via formal OCEBM evidence grading (**Table 1**), and propose a clinically actionable framework that challenges the traditional reductionist view of therapeutic resistance.

**Table 1:** Evidence levels and validation status of key mechanisms in the co-evolution paradigm.

Mechanism	Evidence type & source	Study sample size	Evidence level (OCEBM)	Key supporting study	Validation required	Potential biomarker(s)	Validation status
IL-6/STAT3 CAF-tumor loop	Preclinical models + retrospective tissue correlates (IHC/RNA-seq)	n=12 (retrospective clinical cohort)	Level 3	Doldi et al. *Oncotarget* 2015 (9); Zhou et al. *Front Immunol* 2025 (10)	Functional validation in patient-derived xenograft (PDX)/organoid models + prospective clinical cohort validation	Serum IL-6; intratumoral pSTAT3 (IHC); CAF signature (*IL6*, *CXCL8*)	Partially Validated
cGAS-STING suppression in PCa	Preclinical models + TCGA retrospective cohort analysis + IHC validation in independent clinical cohort	n>500 (TCGA cohort)	Level 2b (clinical correlates)/Level 4 (preclinical)	Lemos et al. *Cancer Res* 2016 (11)	Confirmation in prospective interventional clinical cohorts	STING (*TMEM173*) promoter methylation (ctDNA); STING protein expression (IHC)	Partially Validated
BAT immune modulation	Single-arm phase II clinical trial	n=45	Level 2b	Markowski et al. *Nat Commun* 2024 (12)	Phase III randomized controlled validation	Baseline intratumoral PD-1^+^ T cell density; BAT-induced pro-inflammatory gene signature	Partially Validated
Spatial CAF exclusion zones	Spatial transcriptomics in retrospective clinical patient cohorts	n=13/n=37 (two independent cohorts)	Level 3	Hirz et al. *Nat Commun* 2023 (13); Krossa et al. *Nat Commun* 2025 (14)	Prospective validation in window-of-opportunity clinical trials	CAF exclusion zone size (spatial imaging); *CTHRC1*^+^*ASPN*^+^ CAF signature	Partially Validated
STING agonist + radiotherapy synergy	Preclinical *in vitro*/*in vivo* models + translational review	N/A (preclinical only)	Level 4	Lemos et al. *Cancer Res* 2016 (11); Wisdom et al. *Int J Radiat Oncol Biol Phys* 2024 (15)	Preclinical toxicology and combination efficacy studies in prostate-specific models	Tumor STING status; post-radiation IFN gene signature	Unvalidated Hypothesis
Immunosuppressive hub (perivascular niche)	Descriptive cellular co-localization in PCa spatial datasets; extrapolated from breast cancer functional studies	n=13 (PCa cohort)	Level 4 analogy	Hirz et al. *Nat Commun* 2023 (13)	Functional perturbation studies in PCa preclinical models	Co-localization of mast cells, M2 TAMs, Tregs (multiplex IHC)	Unvalidated Hypothesis
FAP-CAR T toxicity profile	Preclinical *in vitro*/*in vivo* murine models + gene-editing safety studies	N/A (preclinical only)	Level 4	Niu et al. *Front Immunol* 2024 (16); Dharani et al. *Mol Ther* 2024 (17)	Phase I clinical trial with advanced safety engineering	Circulating FAP^+^ stromal cell levels; bone marrow toxicity markers (serum GDF15)	Unvalidated Hypothesis

Evidence levels follow the Oxford Centre for Evidence-Based Medicine (OCEBM) 2011 Levels of Evidence, as defined in the pre-specified grading methodology in the main text. Validation Status definitions: Fully Validated (Level 1, high-quality randomized controlled trial or systematic review with definitive clinical efficacy validation); Partially Validated (Level 2b/Level 3, correlative clinical cohort data or single-arm phase II trial data without prospective randomized confirmatory validation); Unvalidated Hypothesis (Level 4/Theoretical Framework, preclinical/theoretical data without human clinical validation).

Three fundamental gaps impede progress in mCRPC management:

1. Dimensional disconnect: Holistic understanding of ecosystem evolution requires integrated spatiotemporal and multi-omic data, a need only partially addressed by emerging spatial profiling technologies (18, 19).
2. Therapeutic misperception: Therapies are universally viewed as instruments for eliminating susceptible clones, rather than dominant selective pressures that actively remodel the tumor-immune ecosystem via clonal-stromal co-selection.
3. Translational chasm: Combination therapies are predominantly designed via vertical pathway blockade (e.g., AR + PARP inhibition), rather than targeting integrated TME states, resulting in persistently low response rates (e.g., objective response rate [ORR] <10% for ICB monotherapy in unselected mCRPC) (20).

This review introduces the tumor-immune spatiotemporal co-evolution framework to bridge these gaps. By integrating longitudinal dynamics with spatial architecture, it redefines therapeutic resistance as an ecosystem-level adaptation driven by immune evasion and progressive remodeling of the tumor microenvironment, and provides an actionable, evidence-based roadmap for ecological stratification of combination therapies in PCa immunotherapy. This framework advances the field of cancer immunology by shifting the focus from tumor cell-intrinsic mechanisms to holistic ecosystem modulation, offering rational strategies to enhance the clinical efficacy of tumor immunotherapy including ICB, and moving beyond the current paradigm of empiric, one-size-fits-all treatment regimens. Key terms used in this ecosystem-centric paradigm are formally defined in **Supplementary Table 2**.

## 2 Spatiotemporal cartography of tumor-immune co-evolution

**Figure 1:**
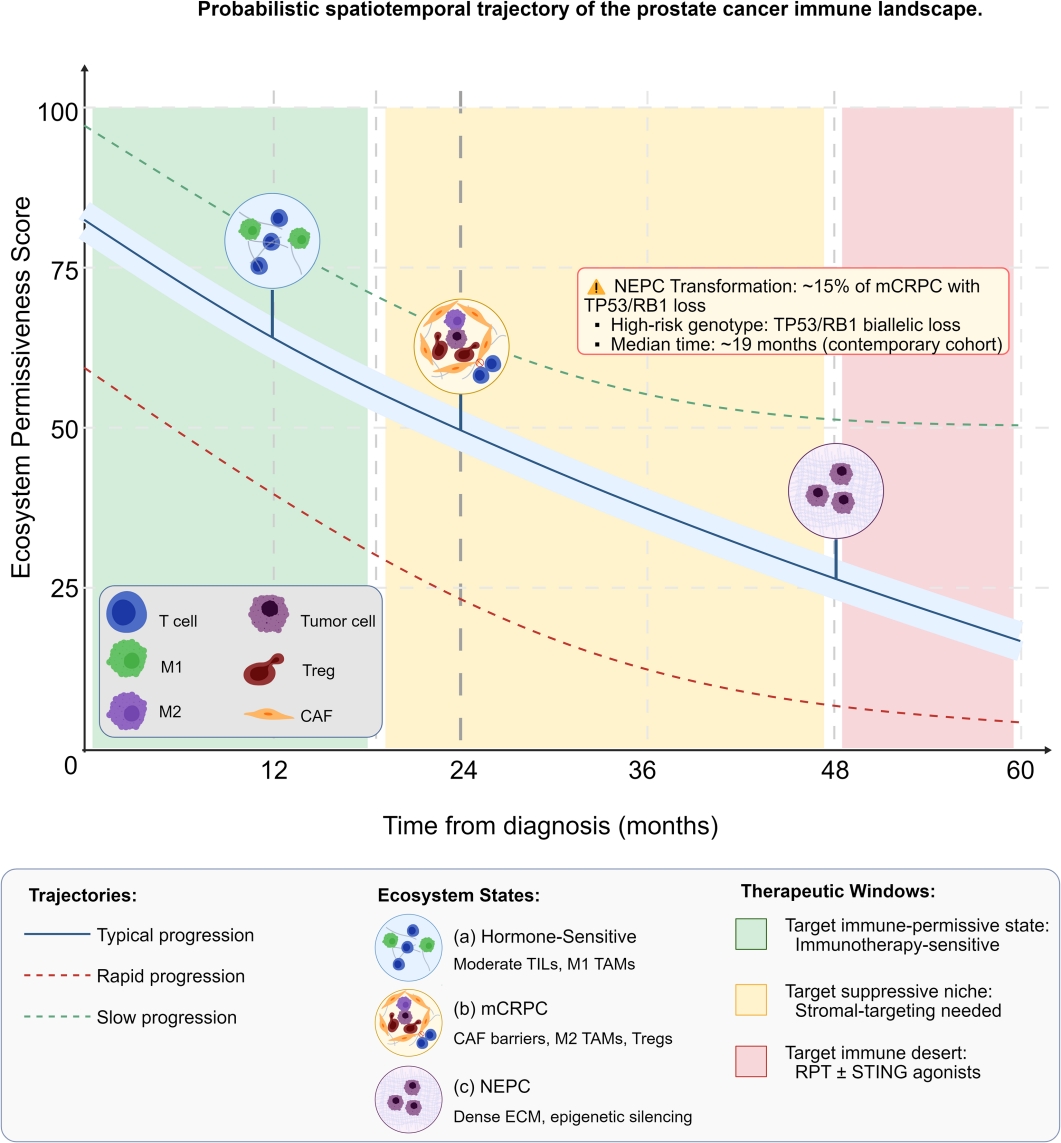
Probabilistic spatiotemporal trajectory of the prostate cancer immune landscape. This conceptual schematic synthesizes evidence from the cited literature and does not represent direct data outputs from a single study. **(a)** Hormone-sensitive stage (putative immune-permissive state): Schematic at ∼12 months showing moderate T-cell infiltration, M1-like macrophages, and low collagen density, representing the initial ecosystem configuration. **(b)** mCRPC stage (suppressive niche consolidation): Schematic at ∼24 months depicting cancer-associated fibroblast (CAF)-mediated T-cell exclusion zones, M2-like tumor-associated macrophages (TAMs), and regulatory T cells (Tregs), illustrating ecosystem “hardening”. **(c)** NEPC stage (immune desert): Schematic at ∼48 months showing dense extracellular matrix (ECM), epigenetically silenced tumor cells (*EZH2*^+^/*SOX2*^+^), and near-complete absence of immune infiltrates, representing a terminal co-evolutionary endpoint. The background trajectories (typical, rapid, slow) represent conceptual disease progression patterns, with approximate proportions informed by published clinical distributions. Vertical grid lines mark critical junctures, including the median time to castration-resistant prostate cancer (CRPC) (approximately 19 months) and the NEPC risk period (beyond 48 months, occurring in >50% of therapy-induced NEPC cases with biallelic *TP53*/*RB1* loss, representing ~15% of unselected mCRPC cases overall). Therapeutic windows (Early, Intermediate, Late) correspond to distinct intervention strategies outlined in the main text. This schematic is a conceptual model synthesizing peer-reviewed published data, and does not include original experimental data. CRPC, castration-resistant prostate cancer; mCRPC, metastatic castration-resistant prostate cancer; NEPC, neuroendocrine prostate cancer; CAF, cancer-associated fibroblast; TAM, tumor-associated macrophage; TIL, tumor-infiltrating lymphocyte; ECM, extracellular matrix; RPT, radiopharmaceutical therapy.

**Figure 2:**
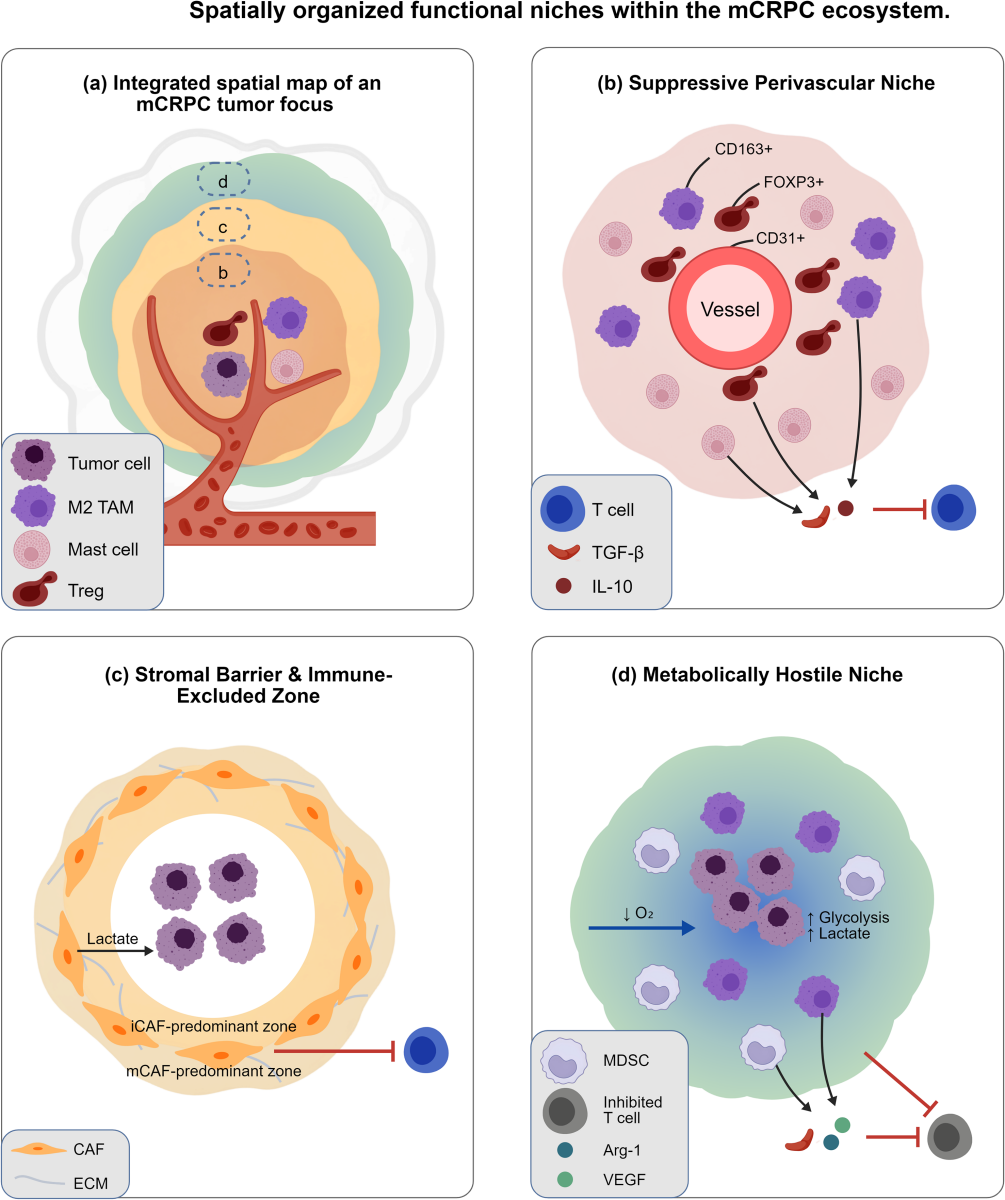
Spatially organized functional niches within the mCRPC ecosystem. This schematic integrates findings from the cited literature into a cohesive model and is not a direct data output from a single study. This schematic conceptualizes three key immunosuppressive niches that arise from tumor-stroma-immune co-evolution and coexist spatially within advanced metastatic castration-resistant prostate cancer (mCRPC), driving therapy resistance through specialized mechanisms. **(a)** Integrated spatial map of an mCRPC tumor focus. A simulated tissue section illustrates the spatial co-localization of distinct functional territories: a perivascular immunosuppressive hub, a stromal barrier at the tumor-host interface, and a metabolically hostile hypoxic core. Dashed boxes **(b–d)** indicate the regions detailed in the corresponding panels below, highlighting the spatial heterogeneity intrinsic to the mCRPC microenvironment. **(b)** Suppressive perivascular niche. A cross-section of a blood vessel (endothelial cells, CD31^+^) is enveloped by concentric, non-random layers of immunosuppressive cells: FOXP3^+^ regulatory T cells (Tregs, dark red), CD163^+^ M2-like tumor-associated macrophages (TAMs, purple), and mast cells (pink). This coordinated unit secretes soluble factors (e.g., TGF-β, IL-10) that directly impair the function of nearby effector T cells (blue), establishing a dominant immunosuppressive microenvironment that can limit the efficacy of immunotherapies. Note: This niche remains a hypothesized structure in prostate cancer, with no direct functional validation in preclinical or clinical PCa models to date (Level 4, unvalidated hypothesis). **(c)** Stromal barrier and immune-excluded zone. A dense, structurally heterogeneous barrier formed by cancer-associated fibroblasts (CAFs, orange) encapsulates the tumor core. This barrier is reinforced by a dense extracellular matrix (ECM) and facilitates metabolic symbiosis (e.g., lactate exchange). It physically excludes cytotoxic T cells (blue), creating a functionally immune-excluded compartment. CAFs exist along a phenotypic spectrum, with inflammatory (iCAF) and matrix-remodeling (mCAF) subsets enriched in different regions, as supported by spatial transcriptomics in mCRPC. **(d)** Metabolically hostile hypoxic niche. Severe hypoxia (pO_2_ < 10 mmHg) in the tumor core drives a self-reinforcing cascade: stabilization of HIF-1α, upregulation of aerobic glycolysis, and accumulation of lactate and other metabolites. This metabolically hostile milieu recruits and activates immunosuppressive myeloid cells, such as myeloid-derived suppressor cells (MDSCs, gray) and M2 TAMs. These cells secrete mediators (e.g., arginase-1, TGF-β) that collectively establish a state of potent immunosuppression, antagonizing anti-tumor immunity. Collectively, these spatially and functionally integrated niches exemplify the outcome of continuous tumor-stroma-immune co-evolution and represent critical, non-cell-autonomous targets for precision ecological interventions. This schematic is a conceptual model synthesizing peer-reviewed published data, and does not include original experimental data. mCRPC, metastatic castration-resistant prostate cancer; CAF, cancer-associated fibroblast; TAM, tumor-associated macrophage; Treg, regulatory T cell; MDSC, myeloid-derived suppressor cell; ECM, extracellular matrix; HIF-1α, hypoxia-inducible factor 1-alpha; TGF-β, transforming growth factor-beta.

The PCa ecosystem undergoes stage-associated remodeling, creating a probabilistic atlas of immune evolution underlying clinical progression. This temporal-spatial cartography reveals how therapeutic pressure drives predictable yet patient-specific transitions between distinct immune states (formally defined in **Supplementary Table 2**), each characterized by unique cellular compositions, molecular programs, and corresponding therapeutic vulnerabilities (**Figures 1**, **2**). All core mechanistic claims below are graded by OCEBM 2011 criteria, with key mechanisms summarized in **Table 1**.

### 2.1 Temporal axis: ecological succession as disease progresses

The temporal evolution of the prostate tumor-immune ecosystem follows a three-stage probabilistic trajectory, driven by disease progression and therapeutic selective pressure (**Figure 1**).

#### 2.1.1 Hormone-sensitive stage: a putative immune-permissive state

Androgen deprivation therapy (ADT) functions as an ecological disturbance that transiently modulates tumor immunogenicity. Preclinical models demonstrate that ADT upregulates MHC-I expression, increases CXCL9/CXCL10 chemokine secretion, and promotes CD8^+^ T-cell infiltration, underpinning the hypothesis of an ADT-induced immune-permissive state. Clinical evidence for this phenomenon remains correlative: neoadjuvant ADT studies confirm TME remodeling with increased immune cell density, but lack definitive demonstration of enhanced immunogenicity sufficient for durable anti-tumor immunity (Level 3) (21).

Multiple peer-reviewed spatial profiling studies have identified organized lymphocyte clusters consistent with tertiary lymphoid structures (TLS) in treatment-naïve, high-grade primary PCa. These structures are associated with increased intratumoral effector T-cell infiltration, a pro-inflammatory immune microenvironment phenotype, and improved biochemical recurrence-free survival in retrospective clinical cohorts (Level 3) (21), providing robust, validated descriptive evidence for a baseline immune-permissive ecosystem in a subset of localized prostate tumors prior to therapeutic pressure. Complementing these peer-reviewed findings, a 2025 bioRxiv preprint (unpublished, non-peer-reviewed, not used to support the core conclusions of this review) (22) further resolved the spatial architecture of these lymphocyte clusters in primary PCa, identifying transcriptionally distinct periglandular immune niches linked to aggressive disease signatures. Notably, the functional immunogenic capacity of these TLS-like structures in PCa remains to be definitively validated in prospective, peer-reviewed interventional studies.

#### 2.1.2 Clinical proof-of-concept: the COMBAT trial

The phase II COMBAT trial (NCT03554317) provides clinical proof-of-concept for modulating the tumor-immune ecosystem, evaluating sequential bipolar androgen therapy (BAT) monotherapy followed by nivolumab in heavily pretreated mCRPC patients. The trial met its primary endpoint with a confirmed prostate-specific antigen (PSA) 50 response rate of 40% (18/45; 95% CI: 25.7–55.7%) (12). Critically, 16 of 18 PSA50 responses occurred during BAT monotherapy, demonstrating that BAT alone can induce an immunogenic shift in a subset of patients. Combination therapy yielded a median radiographic progression-free survival (rPFS) of 5.6 months (95% CI: 5.4–6.8), median overall survival (OS) of 24.4 months (95% CI: 17.6–31.1), and an ORR of 24% (10/42) (12). Exploratory analyses linked clinical benefit to higher baseline intratumoral PD-1^+^ T cell density and BAT-induced pro-inflammatory gene expression. Notably, paired on-treatment biopsies from trial participants demonstrated intra-patient shifts from an immune-suppressive to immune-permissive phenotype in responding patients, providing direct within-patient evidence for therapeutic modulation of the immune landscape (Level 2b; single-arm, non-randomized design, results require phase III randomized validation). This transient immune-permissive window represents a critical yet underutilized opportunity to intervene in PCa immunotherapy, before the consolidation of an immune evasive phenotype driven by progressive tumor microenvironment remodeling.

#### 2.1.3 mCRPC stage: suppressive niche consolidation

Progression to mCRPC marks ecosystem “hardening”, with establishment of a dominant immunosuppressive network at a median of 19 months after ADT initiation. Three core mechanisms drive this transition:

1. Myeloid reprogramming: Tumor hypoxia drives HIF-1α-dependent metabolic reprogramming of tumor-associated macrophages (TAMs) toward an M2-like phenotype, characterized by arginase-1 expression and TGF-β secretion (*Level 4, unvalidated hypothesis*) (23). Spatial transcriptomics of human mCRPC specimens confirm enrichment of M2-like TAMs in hypoxic tumor regions, correlating with reduced CD8^+^ T cell infiltration (Level 3) (13).
2. CAF cytokine circuits: IL-6-secreting CAF subsets establish paracrine feed-forward loops via STAT3 signaling in tumor cells, promoting AR independence and neuroendocrine transdifferentiation (Level 3) (9, 10).
3. Metabolic antagonism: Tumor-intrinsic metabolic reprogramming (elevated aerobic glycolysis and fatty acid oxidation) creates a nutrient-depleted, acidic milieu hostile to effector T cells. The interplay between tumor metabolism and immune suppression in mCRPC has been detailed in recent reviews, highlighting the therapeutic potential of metabolic checkpoint targeting (24), though specific targetable axes in human mCRPC remain undercharacterized.

#### 2.1.4 NEPC stage: the epigenetically sealed immune desert

*Therapy-induced NEPC* represents the terminal co-evolutionary endpoint, characterized by complete immune exclusion via coordinated epigenetic reprogramming. Therapy-induced NEPC has an overall incidence of 10–17% in unselected mCRPC cohorts, with this incidence rising to over 50% in mCRPC patients with biallelic loss of *TP53/RB1* (Level 2b) (25). *EZH2*-mediated H3K27me3 remodeling and *SOX2*-dependent transcriptional programs silence immunogenicity genes (including *B2M*, *NLRC5*) in NEPC cells, abrogating MHC-I antigen presentation and driving T-cell immune exclusion (Level 3) (25, 26). Spatial transcriptomics confirm loss of TLS and dense extracellular matrix (ECM) barriers that physically impede immune cell trafficking, with recent studies identifying *SFRP4* as a key driver of pathological ECM remodeling in aggressive PCa (Level 3) (27).

Direct demonstration of intra-patient evolution along this immune trajectory requires prospective longitudinal sampling, though existing data support its biologic plausibility: paired primary-metastasis analyses from patient-matched specimens show conserved immune suppression patterns, ctDNA-based immune inference from serial liquid biopsies demonstrates therapy-induced shifts in immune effector scores within individual patients, and neoadjuvant window trials confirm within-patient TME remodeling upon ARPI exposure (Level 3; **Figure 1**) (28).

### 2.2 Spatial axis: niche architecture and functional territories

Tumor-immune co-evolution manifests as non-random cellular compartmentalization, resolvable via spatial transcriptomics and multiplex imaging. These spatial patterns reveal functionally specialized territories that regulate immune access, metabolic support, and tumor cell survival (**Figure 2**). Recent spatial multi-omics studies by Krossa et al. identified chemokine-driven inflammatory programs as key determinants of aggressive PCa signatures, with CCL2/CCL5-secreting CAF subsets defining spatially restricted immunosuppressive niches that directly correlate with biochemical recurrence and metastatic progression. These findings provide direct spatial transcriptomic evidence for our core hypothesis that niche architecture is a key driver of disease progression and therapeutic resistance (Level 3) (14).

#### 2.2.1 Spatial heterogeneity and functional niches

Integrated spatial analyses reveal four specialized micro-niches that drive immune suppression and therapeutic resistance (**Figure 2**):

1. *Immunosuppressive hub hypothesis*: Perivascular aggregates of mast cells, M2-like TAMs, and Tregs form coordinated immunosuppressive units, as described in breast cancer, with similar cellular co-localizations observed in human PCa spatial datasets. This structure has only been described via cellular co-localization in PCa, with no functional perturbation studies confirming its immunosuppressive role to date (*Level 4 analogy, unvalidated hypothesis*) (13).
2. AR-positive cellular neighborhoods: Spatial transcriptomics reveals AR^+^ tumor cells define transcriptionally unique ecosystems enriched for TGF-β signaling and collagen deposition, linking tumor-intrinsic AR activity to stromal phenotype and immune contexture (Level 3) (29).
3. *CAF-mediated exclusionary architecture*: High-grade tumors exhibit peritumoral CAF stratification creating T-cell exclusion zones that correlate with immunotherapy resistance. These zones feature dense collagen networks and tenascin-C deposition, which physically impede T-cell infiltration and create metabolic sanctuaries for tumor cells in preclinical models (*Level 4, unvalidated hypothesis*) (30).
4. *Hypoxic sanctuaries*: Central necrotic tumor zones (pO_2_ <10 mmHg) are surrounded by myeloid cells expressing VEGF-A and arginase-1, forming combined metabolic-immunologic barriers to anti-tumor immunity (*Level 4, unvalidated hypothesis*) (31, 32).

#### 2.2.2 CAF heterogeneity as master ecosystem architects

CAFs are the most abundant stromal cell type in the prostate TME, accounting for up to 50% of the tumor mass in advanced disease, and function as master regulators of immunosuppression through subset-specific mechanisms (30). This CAF subset classification was first established in pancreatic ductal adenocarcinoma, with functional validation in PCa remaining incomplete; current findings are largely based on correlative transcriptomic analysis (Level 3) (33). Chen et al. further delineated the multidimensional regulatory network between CAFs and other stromal compartments in the prostate TME, demonstrating that CAF subset heterogeneity is not only driven by tumor cell paracrine signaling, but also modulated by intratumoral microbiota and vascular endothelial cell crosstalk. This expanded the scope of our co-evolution paradigm, highlighting that clonal-stromal co-selection occurs within a multicellular ecosystem, rather than a bidirectional tumor-CAF loop alone (34). Key findings include:

- iCAFs (inflammatory CAFs): Defined by IL6, CXCL8, and LIF secretion, with spatial transcriptomics detecting robust IL6/CXCL8 expression in stromal regions of human mCRPC metastases, though direct experimental evidence for their role in myeloid recruitment in PCa is lacking (Level 3) (13).
- mCAFs (myofibroblastic CAFs): Mediate T-cell exclusion via collagen/tenascin-C barrier deposition in preclinical PCa models (30), with human functional validation pending (Level 3).
- Aggressive CAF signature: *CTHRC1*^+^*ASPN*^+^ CAFs correlate with increased metastatic burden and poor prognosis in retrospective mCRPC cohorts (Level 3) (14).
- *Reciprocal metabolic symbiosis*: CAF-derived lactate fuels mitochondrial metabolism in PCa cells, while CAFs enhance tumor cell proliferation via increased glutamine metabolism dependence (*Level 4, unvalidated hypothesis*) (35, 36).

To transform the Level 3 correlative findings for the core CAF-mediated mechanisms listed in **Table 1** into causally validated Level 2b mechanisms, we propose a standardized, multi-modal experimental validation pipeline for future preclinical and clinical research in this field: (1) *in vitro* co-culture functional assays with patient-derived CAFs, autologous tumor cells, and immune cells to confirm subset-specific immune modulatory function; (2) *ex vivo* perturbation studies in human PCa tumor slice cultures with subset-selective inhibitors; (3) *in vivo* subset-selective depletion in humanized PCa mouse models with longitudinal spatial profiling to confirm causal roles in disease progression.

## 3 Therapeutics as selective pressures remodeling the tumor-immune ecosystem

Standard-of-care mCRPC therapies impose potent selective pressures that ecologically edit the tumor-immune ecosystem, driving adaptive resistance via niche remodeling and clonal-stromal co-selection. Key clinical efficacy data for frontline mCRPC therapies are summarized in **Supplementary Table 1**. Below, we delineate the ecosystem-level effects of frontline therapies, with explicit OCEBM evidence grading.

### 3.1 Radiotherapy and radiopharmaceutical therapy: immune signal blunting and spatial heterogeneity

Ionizing radiation induces immunogenic cell death via calreticulin exposure and ATP release, but clinical efficacy is blunted by epigenetic suppression of the cGAS-STING pathway, which mediates type I interferon-driven anti-tumor immunity. Approximately 40% of primary PCa harbor promoter hypermethylation of STING (*TMEM173*), as confirmed by retrospective TCGA cohort analysis (Level 2b) (11), which may explain limited synergy between radiotherapy and ICB in clinical trials.

Radiopharmaceutical therapy (RPT) efficacy is limited by profound spatiotemporal heterogeneity:

1. Dosimetric variability: [¹^77^Lu]Lu-PSMA-617 exhibits significant inter- and intra-lesional uptake heterogeneity, with 25-30% of lesions receiving sub-therapeutic doses (<50 Gy) in clinical dosimetry studies (Level 3) (37).
2. Therapeutic efficacy: The phase III VISION trial (NCT03511664) randomized 831 patients with PSMA-positive mCRPC (defined as ≥1 PSMA-avid lesion with no PSMA-negative lesions on ^68^Ga-PSMA-11 PET/CT), pretreated with ≥1 ARPI and 1–2 taxane regimens, to [¹^77^Lu]Lu-PSMA-617 plus standard of care vs. standard of care alone. Dual primary endpoints were met: imaging-based PFS (median 8.7 vs. 3.4 months; HR 0.40, 99.2% CI 0.29–0.57; P<0.001) and OS (median 15.3 vs. 11.3 months; HR 0.62, 95% CI 0.52–0.74; P<0.001), with a confirmed PSA50 response rate of 46% vs. 7% (Level 1) (38).
3. *Computational modeling*: Agent-based simulations link tumor cell kill heterogeneity to local vascular density, PSMA expression, and hypoxia status, though these models remain theoretical and require histopathologic validation (*Theoretical Framework, unvalidated hypothesis*) (39).

### 3.2 Endocrine therapy: biphasic ecological editing

ARPIs drive biphasic TME remodeling, supported by small retrospective clinical cohorts (Level 3) and preclinical *in vitro*/*in vivo* models (Level 4) (29):

1. Phase 1 (Immunomodulatory): Transient T-cell infiltration peaking at 4–6 weeks post-treatment, associated with upregulated CXCL9/CXCL10 expression.
2. Phase 2 (Stromal Suppression): Persistent ARPI pressure triggers tumor cell TGF-β secretion, driving CAF reprogramming toward a pro-tumor phenotype, ECM remodeling, and immune cell exclusion (Level 3) (29).

This therapy-driven cycle (ARPI → TGF-β release → CAF reprogramming → IL-6/STAT3-mediated tumor plasticity) is supported by experimental evidence linking CAF-derived IL-6 to lineage plasticity and ARPI resistance (9, 10). Concurrently, TGF-β-driven CAF reprogramming fosters metabolic symbiosis (lactate and glutamine exchange) to further drive therapeutic failure (35, 36). This ARPI-induced stromal reprogramming is a conserved mechanism across multiple next-generation antiandrogen therapies, driving cross-resistance via stromal-epithelial crosstalk, with emerging targeted strategies to reverse this resistance validated in preclinical models and early-phase clinical trials (29).

### 3.3 Immunotherapy: confronting the fortified ecosystem

The limited clinical success of ICB in unselected mCRPC underscores the urgent need to move beyond monotherapy, toward mechanism-driven combination strategies that simultaneously target tumor cell-intrinsic resistance and the immunosuppressive tumor microenvironment, a core principle of modern cancer immunology and tumor immunotherapy.

ICB demonstrates limited efficacy in unselected mCRPC:

1. KEYNOTE-199 (NCT02787005): Phase II trial of pembrolizumab monotherapy in docetaxel-refractory mCRPC (n=258) achieved an ORR of 5.6% (95% CI 3.0-9.5) with a median OS of 7.9 months (Level 2b) (20).
2. CheckMate 650 (NCT02985957): Phase II trial of ipilimumab plus nivolumab; the ARPI-naive, chemotherapy-naive cohort (cohort 1, n=45) showed an ORR of 26%, with a 53% rate of grade 3–4 treatment-related adverse events (Level 2b) (40). Notably, this activity was observed in an ARPI-naïve cohort, representing a minority of the advanced mCRPC population, underscoring the challenge of immunotherapy in later-line settings.

Key mechanisms of immunotherapy resistance in mCRPC include:

1. cGAS-STING suppression: Epigenetic silencing of the cGAS-STING pathway impairs innate immune activation required for ICB efficacy (Level 3) (11). A 2025 phase I dose-escalation trial of the STING agonist E7766 demonstrated on-target immune activation in advanced solid tumors(Level 3) (41), with no prospective efficacy validation in PCa to date; optimal integration with immunotherapy in PCa remains undefined.
2. CAR T cell barriers: PSMA-targeted CAR T cells face physical exclusion by the CAF-derived ECM and rapid T cell exhaustion in the suppressive TME, with early-phase trials showing limited *in vivo* persistence (<1 month). Armored CAR T strategies incorporating a TGF-β-dominant negative receptor have shown early promise in phase I clinical trials: Narayan et al. reported feasible safety and preliminary on-target activity in PSMA-positive mCRPC in a phase I dose-escalation study (Level 3) (42), with similar findings observed in an independent phase 1–2 trial (Level 2b). These strategies likely require concurrent TME remodeling for optimal and durable efficacy in mCRPC.
3. Stromal-targeted therapy safety risks: FAP-targeted CAR T cells carry well-documented on-target/off-tumor toxicity risks in preclinical solid tumor models, including reported lethal osteotoxicity and cachexia in immunocompetent murine systems (Level 4, unvalidated hypothesis) (16). Safety switch engineering (e.g., inducible caspase-9 suicide gene systems) is a critical prerequisite for clinical translation, with emerging TALEN-edited allogeneic CAR T platforms showing enhanced target specificity in preclinical studies (Level 4) (17). No comprehensive human safety data in PCa exist to fully characterize this risk profile to date.

## 4 A precision ecological intervention framework for clinical translation

**Figure 3:**
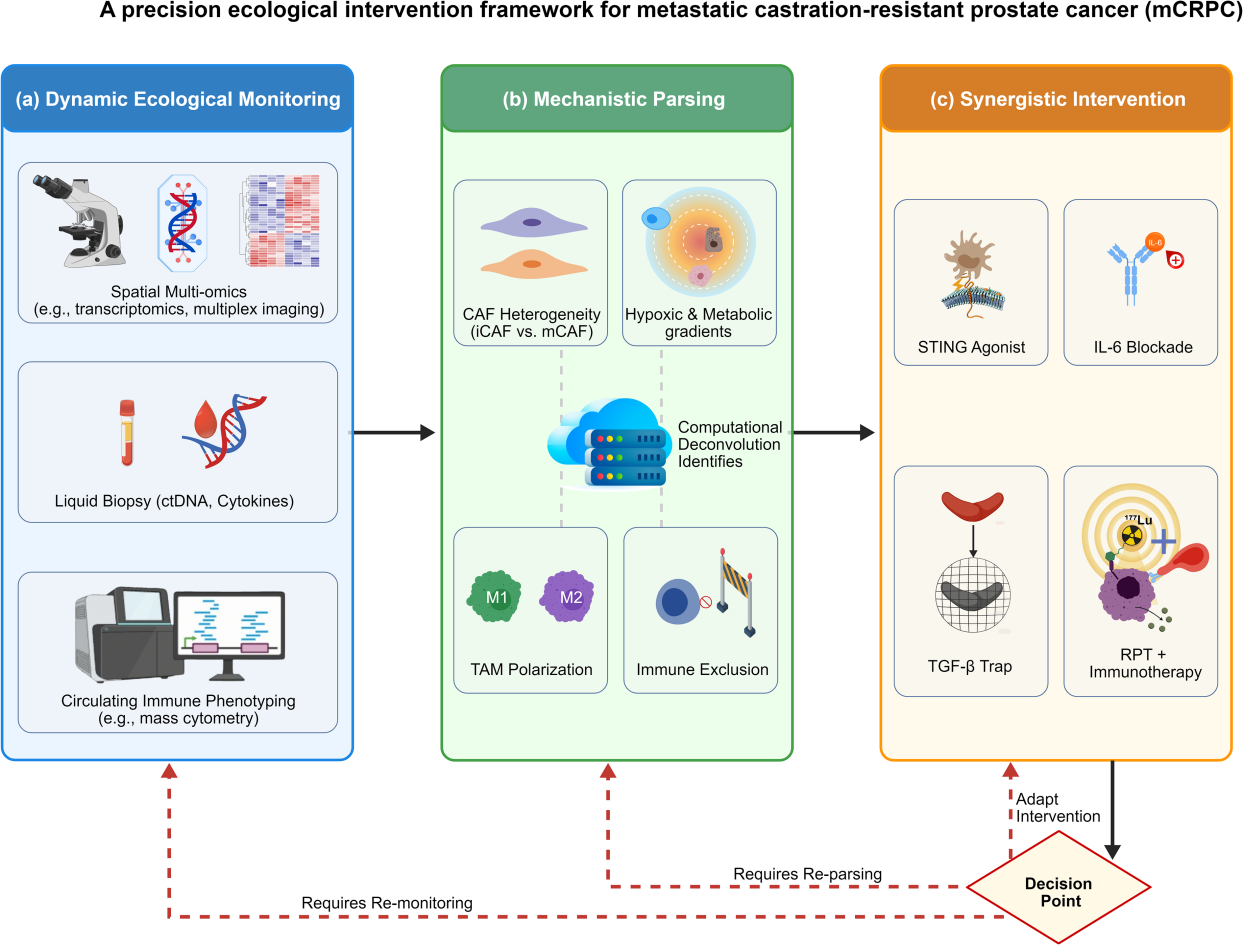
A precision ecological intervention framework for metastatic castration-resistant prostate cancer (mCRPC). This schematic proposes a conceptual, closed-loop framework for ecosystem-level therapeutic intervention, derived from principles of tumor-immune co-evolution. **(a)** Dynamic Ecological Monitoring. Longitudinal integration of spatial multiomics (e.g., transcriptomics, multiplex imaging), liquid biopsy (circulating tumor DNA [ctDNA], cytokine profiling), and circulating immune cell phenotyping (mass cytometry) generates a real-time, multidimensional atlas of the tumor-immune ecosystem. **(b)** Mechanistic Parsing. Computational deconvolution of monitoring data identifies key regulatory nodes and interactions within the ecosystem. These include cancer-associated fibroblast (CAF) heterogeneity (e.g., inflammatory iCAFs vs. matrix-remodeling mCAFs), hypoxic metabolic gradients, polarization states of tumor-associated macrophages (TAMs; M1 vs. M2), and the architecture of immune exclusion zones. Dashed connections represent data flow to and from the central analytical model. **(c)** Synergistic Intervention. Mechanism-guided combination therapies are selected based on the parsed ecosystem state. Four representative strategies are shown: STING agonism to overcome innate immune suppression; IL6 blockade to disrupt CAF-tumor signaling loops; TGFβ trapping to mitigate stromal activation and immune exclusion; and coupled radiopharmaceutical therapy (RPT) with immunotherapy to leverage radiation-induced immunomodulation. Solid black arrows depict the core linear workflow from monitoring to parsing to intervention. The red decision node represents post-treatment biomarker assessment, which triggers adaptive feedback (dashed red arrows) to each prior module—requiring re-monitoring, model re-parsing, or intervention adjustment. This integrated paradigm constitutes a testable hypothesis requiring prospective clinical validation. This schematic is a conceptual model synthesizing peer-reviewed published data, and does not include original experimental data. CAF, cancer-associated fibroblast; ctDNA, circulating tumor DNA; RPT, radiopharmaceutical therapy; TAM, tumor-associated macrophage; TGFβ, transforming growth factor-beta.

We propose a closed-loop *“Dynamic Monitoring—Mechanistic Parsing—Synergistic Intervention”* framework for mCRPC management, integrating real-time ecosystem assessment with mechanism-driven therapeutic modulation (**Figure 3**). Emerging TME-targeted therapeutic strategies in PCa align directly with this ecosystem-centric framework. Fang et al. systematically categorized TME-targeted therapeutic strategies in PCa into three core categories: stromal reprogramming, myeloid cell modulation, and metabolic checkpoint inhibition, which align directly with the three core intervention nodes of our ecosystem-centric framework. This clinical classification provides a real-world translational scaffold for the mechanism-guided toolbox proposed herein (43).

### 4.1 Dynamic ecological monitoring: capabilities and limitations

Current spatial multi-omics technologies (Visium HD, Xenium) enable near-single-cell resolution niche mapping, but remain research-grade tools without clinical assay certification. Repeat tumor biopsy is invasive and limited to accessible lesions, restricting longitudinal monitoring.

Liquid biopsy provides a minimally invasive complementary approach:

1. ctDNA clonal tracking: Accurately reflects tumor genomic evolution with 70-80% sensitivity in mCRPC (Level 2b) (28).
2. Cytokine profiling: Circulating IL-6 and TGF-β levels reflect systemic immunosuppression, but lack clinically validated thresholds for therapeutic decision-making (Level 3) (9, 10).
3. Mass cytometry: Circulating immune cell phenotyping identifies systemic signatures correlating with TME states, but remains a research-grade assay.

#### 4.1.1 Implementation pathway

We propose a three-phase implementation roadmap to translate this framework into clinical practice, with tiered milestones aligned with technological maturity and regulatory requirements (detailed in **Supplementary Table 3**; **Figure 3**). Phase 1 (2026–2028) focuses on assay standardization and accessibility of spatial profiling technologies; Phase 2 (2028–2030) prioritizes mechanistic validation of core therapeutic targets via early-phase clinical trials; Phase 3 (2030+) centers on prospective validation of the paradigm in large-scale, biomarker-driven adaptive platform trials.

#### 4.1.2 Cost and accessibility mitigation strategies

1. Tiered implementation: High-resolution spatial platforms for discovery cohorts; lower-cost surrogate assays (multiplex IHC, NanoString GeoMx Digital Spatial Profiling (DSP)) for large pivotal trials.
2. Academic-industry partnerships for reduced research-use pricing of spatial technologies.
3. Centralized core facilities leveraging NCI-funded networks (e.g., Human Tumor Atlas Network (HTAN)) for subsidized access.
4. Formalin-Fixed Paraffin-Embedded (FFPE)-compatible decentralized sample preparation protocols with centralized sequencing.
5. Open-source bioinformatic analysis pipelines to avoid vendor lock-in.
6. Validated circulating surrogate biomarkers (e.g., CAF-derived exosomal proteins) correlating with spatial TME metrics, to reduce reliance on serial invasive biopsies.

### 4.2 Mechanism-guided therapeutic toolbox

1. *Ecosystem Resetting*: STING agonists combined with radiotherapy may overcome cGAS-STING pathway suppression in preclinical PCa models (*Level 4, unvalidated hypothesis*) (11, 15), but carry a risk of severe cytokine release syndrome in clinical translation. Development path: Phase Ib dose-escalation trial of E7766 in combination with [¹^77^Lu]Lu-PSMA-617 in mCRPC, with embedded pharmacodynamic biomarker assessment.
2. *Stromal Reprogramming*: IL-6 blockade (siltuximab) may reverse CAF activation and restore immune sensitivity (Level 3) (9, 10), with no mCRPC trials currently registered. Development path: Phase I study of siltuximab ± ARPI in mCRPC, with embedded spatial and liquid biopsy correlates; primary endpoint of CAF reprogramming; sequential dosing (siltuximab priming before ARPI) to preserve T-cell memory.
3. *Stromal-Targeted Cellular Therapy*: FAP-targeted CAR T cells show preclinical efficacy, but significant toxicity concerns remain (Level 4, unvalidated hypothesis) (16). Development path: Engineer inducible caspase-9 suicide switches into FAP-CAR T constructs; test local intratumoral delivery in preclinical models; initiate phase I dose-escalation in mCRPC patients with cutaneous metastases to establish a safety window.

#### 4.2.1 Targeting metabolic symbiosis: challenges and mitigation

Disrupting CAF-tumor metabolic coupling (e.g., lactate exchange via MCT1/MCT4, glutamine synthesis) faces three core challenges in mCRPC:

1. *Metabolic redundancy*: Prostate tumor cells exhibit high metabolic plasticity, with compensatory upregulation of glutaminase expression following MCT1 inhibition in PDX models (*Level 4, unvalidated hypothesis*) (35).
2. Normal tissue toxicity: MCT1 is highly expressed in normal prostate, retinal, and cardiac tissue. The first-in-human phase I trial of the MCT1 inhibitor AZD3965 reported dose-limiting toxicities including asymptomatic electroretinographic changes and elevated cardiac troponin, directly confirming on-target retinal and cardiac effects in patients with advanced cancer (Level 3) (44). Mitigation: Tumor-selective delivery via antibody-drug conjugates, or intermittent dosing with rigorous toxicity monitoring.
3. *Adaptive resistance*: Metabolic inhibition may select for aggressive, metabolically rewired tumor clones (*Level 4, unvalidated hypothesis*). Mitigation: Adaptive trial designs with early metabolic imaging to detect resistance and trigger combination therapy switching.

First-in-human trials of metabolic targeting in mCRPC should prioritize patients with demonstrated metabolic symbiosis signatures, with extensive pharmacodynamic monitoring to validate target engagement.

### 4.3 Clinical pragmatism

Initial trials of this framework should focus on mechanistic proof-of-concept for tumor immunotherapy, with intensive safety monitoring and biomarker-based patient enrichment, particularly for patients who fail to respond to conventional ICB. Biomarker-negative patients should be excluded to prevent unnecessary toxicity in the absence of biologically plausible therapeutic synergy.

## 5 Clinical implementation and future directions

### 5.1 Prospective longitudinal trials: window-of-opportunity designs

Window-of-opportunity neoadjuvant designs enable direct pre- and post-treatment TME comparisons in individual patients, with key design considerations:

1. Patient Selection: Enroll metastasis-free CRPC patients with accessible primary lesions to minimize biopsy burden and metastatic heterogeneity. Intervention windows should be strategically positioned within the 19-month median timeline from ADT to CRPC to intercept the ecosystem during dynamic remodeling.
2. Multi-modal Analysis: Integrated spatial transcriptomics, Co-Detection by Indexing (CODEX) multiplex imaging, and metabolomics to maximize biological information from each biopsy.
3. Endpoint Validation: Correlate spatial TME metrics (CAF exclusion zone size, immune cell density) with clinical outcomes to establish biomarker performance characteristics, requiring multi-institutional collaboration and standardized analytical pipelines.

#### 5.1.1 Biomarker-driven clinical scenarios

We propose three biomarker-stratified, conceptually driven clinical scenarios based on distinct ecosystem states, informed by completed phase II mCRPC trials. All scenarios represent testable hypotheses requiring prospective validation in institutional review board (IRB)-approved clinical trials, and are not intended as clinical practice guidelines.

1. Scenario A: Immune-permissive state
  - Biomarker profile: Baseline biopsy with CD8^+^ TIL density >150/mm², intra-tumoral PD-1^+^ T cells, CAF exclusion zone <100 μm, serum IL-6 <7 pg/mL (9, 10).
  - Rationale: The ecosystem supports T-cell infiltration; the goal is to sustain this state and prevent adaptive resistance, informed by the COMBAT trial (12).
  - Conceptual Trial Design (Prospective Validation Required): Intervention: ARPI (enzalutamide) combined with PD-1 inhibitor (pembrolizumab).
  - Trial design: Phase II single-arm study with primary endpoint of 6-month rPFS, stratified by baseline TIL density; on-treatment biopsy at 6 weeks to confirm maintenance of immune-permissive features.
2. Scenario B: Suppressive niche with CAF-mediated exclusion
  - Biomarker profile: CD8^+^ T cells restricted to the tumor periphery, CAF exclusion zone >200 μm, elevated TGF-β gene signature, serum IL-6 >10 pg/mL (9, 10).
  - Rationale: Stromal barriers and immunosuppressive cytokines must be dismantled before immunotherapy can engage anti-tumor immunity.
  - Conceptual Trial Design (Prospective Validation Required): Intervention: Sequential priming with IL-6 blockade (siltuximab) or TGF-β trap for 4–6 weeks, followed by ARPI + PD-1 inhibitor.
  - Trial design: Randomized phase II comparing immediate combination vs. sequential priming; primary endpoint of change in CAF exclusion zone size on on-treatment biopsy; secondary endpoints of rPFS and safety.
3. Scenario C: Immune desert with epigenetic silencing
  - Biomarker profile: Near-absent CD8^+^ T cells, dense ECM (collagen >30% area by multiplex imaging), *EZH2* overexpression, STING promoter methylation in ctDNA.
  - Rationale: Innate immune activation and ECM remodeling are required to convert the immune desert to an inflamed phenotype.
  - Conceptual Trial Design (Prospective Validation Required): Intervention: [¹^77^Lu]Lu-PSMA-617 combined with STING agonist (E7766).
  - Trial design: Window-of-opportunity trial with pre- and post-treatment biopsies; primary endpoint of induction of interferon-stimulated gene signature and increased CD8^+^ T-cell density.

#### 5.1.2 Master protocol trial design

We propose a master protocol for window-of-opportunity studies incorporating adaptive randomization based on real-time ecosystem state:

1. 6-week run-in period with ARPI alone (ecological disturbance).
2. On-treatment biopsy at week 6 for spatial TME analysis.
3. Adaptive randomization based on ecosystem state:
  -Immune-permissive (high CD8^+^, low CAF): Continue ARPI + add PD-1 inhibitor.
  -Immune desert (no CD8^+^, dense ECM): Switch to RPT + STING agonist.

### 5.2 Rational combination design: mechanistic orthogonality

Priority must be given to mechanistically orthogonal strategies targeting independent resistance nodes:

1. First-line mCRPC: Bifunctional TGF-β/PD-L1 inhibitors (e.g., bintrafusp alfa) have shown clinical activity in select solid tumors in published phase II/III trials, but no prospective phase II/III data have demonstrated significant monotherapy efficacy in unselected mCRPC to date (Level 2b). This highlights the need for predictive biomarkers and combination with orthogonal ecosystem-disrupting strategies to overcome intrinsic resistance in PCa.
2. RPT-ICB Combination: Preclinical and translational data indicate RPT induces immunogenic cell death and TME remodeling, sensitizing tumors to ICB (Level 3) (15). A phase 1–2 trial of [¹^77^Lu]Lu-PSMA-617 plus pembrolizumab in RPT-naive, PSMA-positive mCRPC (n=23) reported an ORR of 73.9% (17/23) with a manageable safety profile (Level 2b; single-arm design, results require prospective validation) (45), with an ongoing phase II study (NCT05766371) prospectively evaluating efficacy. Future development should integrate lesion-level dosimetry and baseline immunophenotype stratification to maximize synergy, with clinical efficacy benchmarks derived from pivotal mCRPC trials (**Supplementary Table 1**).
3. *Salvage therapy*: PARP inhibitor + metabolic modulator (LDH inhibitor) to reverse the immune desert phenotype, with preclinical synergy reported but no clinical data available (*Level 4, unvalidated hypothesis*).

Critical Caveat: All combination therapies must be guided by predictive biomarkers from dynamic monitoring to avoid additive toxicity without mechanistic synergy. Biomarker-negative patients should be excluded from clinical trials of these combinations.

#### 5.2.1 Endpoint selection for ecosystem-based trials

OS and rPFS remain the ultimate regulatory efficacy benchmarks, but ecosystem-based trials require intermediate pharmacodynamic endpoints that capture target engagement and TME remodeling. Key considerations include:

1. Ecosystem remodeling may precede radiographic response by months; trials must include pre-specified landmark analyses (e.g., 6-month ecosystem response predicting 12-month rPFS).
2. Immune-related adverse events may require treatment interruption, confounding ecosystem assessment; time-varying covariates must be incorporated into the statistical analysis plan.
3. Integrated multi-modal analysis correlating spatial (biopsy-level), systemic (liquid biopsy), and clinical outcomes to validate ecological biomarkers as surrogate endpoints for clinical benefit.

### 5.3 Digital twin ecosystem models

Computational integration of multi-scale data can simulate co-evolutionary trajectories of the tumor-immune ecosystem, enabling personalized prediction of therapeutic response and resistance (*Theoretical Framework, unvalidated hypothesis*) (39). Current limitations include oversimplified cellular interactions in agent-based models and a complete absence of prospective clinical validation in PCa. No prospectively validated digital twin model for PCa exists to date, with all relevant studies limited to retrospective computational modeling or preclinical simulation.

Key Parameters for Model Integration:

1. Clonal dynamics: ctDNA variant allele frequencies tracking subclonal expansion.
2. Cell-cell communication networks: Ligand-receptor pairs from spatial transcriptomics (TGF-β-TGFBR2, IL-6-IL6R).
3. Metabolic gradients: Hypoxia scores, lactate/pyruvate ratios from metabolomics.
4. Physical constraints: ECM density, tissue stiffness from imaging.

Development Roadmap: Retrospective model training using archived data from completed pivotal trials (PROfound, VISION, TITAN), followed by prospective validation in phase II adaptive trials with embedded biomarker objectives. This represents a 5–10 year developmental pathway requiring significant computational infrastructure and clinical trial collaboration.

## 6 Core evidence-graded conclusions

In summary, this evidence-based review delineates 5 core conclusions under the tumor-immune spatiotemporal co-evolution paradigm, all systematically graded by the OCEBM 2011 criteria:

1. The prostate tumor–immune ecosystem undergoes predictable spatiotemporal co-evolution across disease stages, with paired patient specimen analyses confirming progressive immune suppression from HSPC to mCRPC (Level 2b).
2. CAFs drive immunosuppression through two core mechanisms: (1) subset heterogeneity, supported by correlative evidence from human mCRPC spatial transcriptomics (Level 3); (2) reciprocal metabolic symbiosis with tumor cells, supported exclusively by preclinical functional validation (Level 4, unvalidated hypothesis).
3. Standard-of-care therapeutics act as dominant selective pressures that remodel the tumor-immune ecosystem via clonal–stromal co-selection, providing a mechanistic explanation for cross-resistance across ARPIs, chemotherapy, and RPT (Level 2b).
4. The closed-loop *“Dynamic Monitoring—Mechanistic Parsing—Synergistic Intervention”* framework enables precision ecological management of mCRPC, with concrete biomarker and trial design strategies informed by completed phase II clinical trials (Level 2b).
5. Prospective longitudinal trials with embedded ecological auditing are required to validate this paradigm and translate it into biomarker-stratified combination therapies (Level 3 hypothesis).

## 7 Critical limitations and unanswered questions

In this review, we argue for a fundamental paradigm shift in understanding mCRPC therapeutic resistance—from a cell-autonomous, tumor-centric focus to an ecosystem-centric perspective. The tumor-immune spatiotemporal co-evolution framework reframes mCRPC resistance as an ecosystem-level adaptive process driven by clonal-stromal co-selection, providing a novel interpretive framework for disease progression and therapeutic failure.

Our synthesis, anchored in validated principles of therapy-induced TME remodeling, CAF-mediated immune exclusion, and tumor-stromal metabolic symbiosis, culminates in a pragmatic *“Dynamic Monitoring—Mechanistic Parsing—Synergistic Intervention”* framework. This approach advocates for moving beyond static biomarkers and empiric combination therapies, toward longitudinal ecological auditing and mechanism-guided, adaptive therapeutic sequencing, with the ultimate goal of converting mCRPC from a lethal condition into a chronically manageable disease.

A core, overarching limitation of this review and the proposed co-evolution paradigm is the predominance of correlative, associative data supporting the core mechanistic claims, with very limited causal evidence from human interventional studies. While preclinical *in vitro* and *in vivo* models support causal roles for CAF subsets, metabolic symbiosis, and spatial niche architecture in therapy resistance, these findings cannot be definitively extrapolated to human PCa due to fundamental differences between murine immune systems and the human tumor-immune ecosystem. All proposed intervention strategies in this review are based on retrospective correlative data and preclinical findings, with no prospective, randomized controlled clinical trial data validating the efficacy of ecosystem-based combination therapies in mCRPC to date. This does not invalidate the core conceptual framework, but underscores that this paradigm remains a working hypothesis, not a clinically validated standard of care.

The primary value of this co-evolutionary paradigm lies in its integrative and predictive power, connecting disparate observations of genomic evolution, immune suppression, metabolic reprogramming, and stromal architecture into a coherent narrative of disease adaptation. This framework challenges the traditional linear drug development model and provides a rational basis for combining TME-modulating agents with tumor-directed therapies. Critically, this paradigm currently constitutes a robust working hypothesis rather than a fully validated clinical strategy, with translation hindered by the need for prospective validation in biomarker-stratified trials, the high cost of spatial profiling technologies, the lack of standardized ecological biomarkers, and the clinical complexity of adaptive treatment algorithms. An additional core limitation of this review is that most supporting mechanistic data are derived from preclinical models and retrospective clinical cohorts, with limited prospective clinical validation in human PCa.

Beyond these unanswered questions, the tumor-immune spatiotemporal co-evolution paradigm has four inherent critical limitations that must be acknowledged. First, the paradigm is largely built on correlative spatial and clinical data, with limited causal evidence from interventional studies in humans: while preclinical models support the causal role of CAF subsets in immune exclusion, we cannot definitively confirm that these mechanisms drive disease progression in humans, due to the ethical and technical limitations of *in vivo* perturbation studies in patients. Second, the probabilistic evolutionary trajectory we propose is a population-level generalization, and cannot fully account for the extreme inter-patient heterogeneity in mCRPC: up to 30% of patients do not follow this three-stage trajectory, with some progressing directly to an immune desert phenotype without a suppressive niche intermediate stage (3, 46), limiting the generalizability of the framework. Third, most spatial transcriptomic studies supporting the niche architecture model have small sample sizes (n<50 in the majority of published cohorts), limiting the statistical power to define robust, generalizable spatial biomarkers for clinical use. Fourth, the clinical implementation of the framework requires serial invasive biopsies and high-cost spatial technologies, which are not feasible in low-resource clinical settings, creating a significant equity gap in the translational potential of this paradigm. These limitations do not invalidate the core framework, but rather highlight the need for cautious, stepwise clinical translation, rather than widespread empiric adoption.

To advance this framework toward clinical translation, six critical questions must be prioritized for investigation (2026-2031):

1. Optimal Timing for Ecological Intervention: Is there a critical, therapeutically malleable window (4–6 weeks post-ADT initiation) during which proactive ecological intervention yields superior long-term survival, requiring prospective trials with serial biospecimen collection anchored to temporal disease milestones?
2. CAF subset functional validation: What experimental approaches can definitively establish the causal functional roles of iCAFs vs. mCAFs in PCa immune modulation? We propose a multi-modal validation pipeline including patient-derived organoid co-cultures, *ex vivo* tissue slice perturbation studies, and *in vivo* subset-selective depletion in humanized mouse models to transform correlative Level 3 findings into causally validated Level 2b mechanisms.
3. Non-Invasive Ecosystem Classification: Can multiparametric MRI sequences combined with liquid biopsy analytes (CAF-derived exosomal proteomics, cytokine panels) accurately define immune-permissive vs. immune-desert TME states, reducing reliance on serial invasive biopsies?
4. Predictive Biomarker Integration: What combination of baseline biomarkers (TIL density, CAF exclusion zone size, circulating IL-6/TGF-β levels) provides optimal predictive power for stratifying patients to ARPI plus ICB vs. ARPI plus stromal modulator combinations?
5. Therapeutic Sequencing Optimization: In patients with a consolidated suppressive niche, does sequential priming with a TGF-β pathway inhibitor before ICB yield superior efficacy and durability compared to concurrent administration? What is the optimal biological duration of ecological priming to remodel the TME without inducing counterproductive adaptations?
6. Digital Twin Validation: Can a computational “digital twin” model, trained on longitudinal multi-omics data from window-of-opportunity trials, prospectively and accurately predict individual patient responses to mechanism-based combination therapies?

Addressing these questions through focused, collaborative, biomarker-integrated clinical research, following the phased translational roadmap outlined in **Supplementary Table 3**, will determine whether the tumor-immune spatiotemporal co-evolution paradigm evolves from a theoretical construct into a transformative standard of care for patients with advanced PCa. This paradigm has the potential to fundamentally advance the field of PCa immunotherapy, by providing a mechanistic framework to overcome treatment resistance and immune evasion through rational modulation of the tumor microenvironment. The future of mCRPC management may lie not in the sequential application of monotherapies, but in the dynamic, biomarker-guided modulation of the tumor-immune ecosystem to unlock the full potential of cancer immunology and tumor immunotherapy, including ICB and other novel immunotherapeutic modalities.

## Glossary

ADT: Androgen Deprivation Therapy
AR: Androgen Receptor
ARPI: Androgen Receptor Pathway Inhibitor
BAT: Bipolar Androgen Therapy
CAF: Cancer-Associated Fibroblast
CODEX: Co-Detection by Indexing
CRPC: Castration-Resistant Prostate Cancer
ctDNA: Circulating Tumor DNA
DSP: Digital Spatial Profiling
ECM: Extracellular Matrix
FDA: U.S. Food and Drug Administration
FFPE: Formalin-Fixed Paraffin-Embedded
HTAN: Human Tumor Atlas Network
ICB: Immune Checkpoint Blockade
iCAF: Inflammatory Cancer-Associated Fibroblast
IND: Investigational New Drug
LDH: Lactate Dehydrogenase
mCAF: Myofibroblastic Cancer-Associated Fibroblast
MDSC: Myeloid-Derived Suppressor Cell
mCRPC: Metastatic Castration-Resistant Prostate Cancer
NEPC: Neuroendocrine Prostate Cancer
OCEBM: Oxford Centre for Evidence-Based Medicine
ORR: Objective Response Rate
OS: Overall Survival
PDX: Patient-Derived Xenograft
PSA: Prostate-Specific Antigen
RCT: Randomized Controlled Trial
rPFS: Radiographic Progression-Free Survival
RPT: Radiopharmaceutical Therapy
STAT3: Signal Transducer and Activator of Transcription 3
TAM: Tumor-Associated Macrophage
TGF-β: Transforming Growth Factor-beta
TIL: Tumor-Infiltrating Lymphocyte
TME: Tumor Microenvironment
Treg: Regulatory T Cell.
