# Supplementary material for: Tumor-immune spatiotemporal co-evolution as a paradigm for overcoming therapy resistance in advanced prostate cancer
Source: Front Immunol. 2026 Mar 11;17:1797299. doi: 10.3389/fimmu.2026.1797299 (PMC13013517; doi:10.3389/fimmu.2026.1797299)
Supplement: Supplementary file 1 [file Table1.docx]

**Supplementary Table 1 | Summary of pivotal clinical trials in mCRPC**

| **Trial** | **Phase** | **N** | **Intervention** | **Primary Endpoint** | **PSA50 Response** | **Key Efficacy Outcomes** |
| --- | --- | --- | --- | --- | --- | --- |
| COMBAT | II | 45 | Sequential BAT → Nivolumab | Confirmed PSA50 response rate | 40% (18/45) | Median rPFS: 5.6 mo (95% CI: 5.4–6.8); Median OS: 24.4 mo (95% CI: 17.6–31.1); ORR: 24% (10/42) |
| TheraP | II | 291 | [¹⁷⁷Lu]Lu-PSMA-617 vs Cabazitaxel | PSA-PFS | 66% vs 37% | Median PSA-PFS: 5.8 vs 5.4 mo (HR 0.67) |
| VISION | III | 831 | [¹⁷⁷Lu]Lu-PSMA-617 + SOC vs SOC | Imaging-based PFS & OS (dual primary) | 46% vs 7% | OS (n=831): Median 15.3 vs 11.3 mo (HR 0.62, 95% CI 0.52–0.74); rPFS (n=581): Median 8.7 vs 3.4 mo (HR 0.40, 99.2% CI 0.29–0.57) |
| KEYNOTE-199 | II | 258 | Pembrolizumab | ORR (Cohort 1) | 5.6% | Median OS: 7.9 mo |
| CheckMate 650 | II | 90 | Ipilimumab + Nivolumab (cohort 1 & 2) | ORR | 26% (cohort 1) | Median OS (cohort 1): 8.2 mo; Grade 3–4 AEs: 53% |

*Note: All data are derived from published peer-reviewed clinical trials as cited in the main text. Abbreviations: mCRPC, metastatic castration-resistant prostate cancer; BAT, bipolar androgen therapy; PSA, prostate-specific antigen; rPFS, radiographic progression-free survival; OS, overall survival; ORR, objective response rate; SOC, standard of care; AEs, adverse events.*

**Supplementary Table 2 | Key Definitions in Ecological Oncology**

| **Term** | **Definition** |
| --- | --- |
| Tumor-Immune Co-evolution | Reciprocal adaptation between malignant clones and immune-stromal ecosystems under therapeutic selection pressure, analogous to predator-prey dynamics with clonal-stromal co-selection. |
| Immune-Permissive State | TME configuration allowing effector T-cell infiltration and function, characterized by M1-polarized macrophages, low TGF-β signalling, intact antigen presentation, and minimal ECM barriers. |
| Suppressive Niche Consolidation | Process by which therapeutic pressure selects for stromal and myeloid populations that actively exclude and inhibit anti-tumor immunity through metabolic antagonism and physical barriers. |
| Immune Desert | Terminal state of immune exclusion mediated by dense ECM deposition, epigenetic silencing of immunogenicity genes, and loss of tertiary lymphoid structures. |
| Clonal-Stromal Co-selection | Simultaneous evolution of tumor subclones and CAF populations that mutually support survival under therapeutic pressure through reciprocal signalling and metabolic symbiosis. |

*Abbreviations: TME, tumor microenvironment; TGF-β, transforming growth factor-beta; ECM, extracellular matrix; CAF, cancer-associated fibroblast.*

**Supplementary Table 3 | Translational Roadmap: From Monitoring to Intervention**

| **Phase** | **Timeline** | **Core Activities** |
| --- | --- | --- |
| Phase 1: Dynamic Monitoring | 2026-2028 | 1. Standardize clinical-grade liquid biopsy assays for ctDNA clonal tracking and cytokine panels (IL-6, TGF-β). Define reference ranges in treatment-naïve vs. treated mCRPC.  2. Implement research-grade spatial transcriptomics in multi-institutional window-of-opportunity trial designs. Develop consensus pipelines for CAF and immune cell quantification.  3. Negotiate academic-industry partnerships for spatial technology access at reduced cost. Prioritize high-yield patients (metastasis-free CRPC with accessible primaries). |
| Phase 2: Mechanistic Validation | 2028-2030 | 1. Initiate phase I studies of IL-6 blockade (siltuximab) ± ARPI with embedded spatial and liquid biopsy correlates. Primary endpoint: CAF reprogramming (collagen reduction, iCAF→mCAF shift).  2. Conduct phase Ib trials of [¹⁷⁷Lu]Lu-PSMA + pembrolizumab in high-TIL patients to test synergy hypothesis. Use dosimetry to stratify lesions.  3. Develop and validate inducible caspase-9 suicide switches for FAP-CAR T in *in vitro* bone marrow models before Investigational New Drug (IND) filing. |
| Phase 3: Adaptive Trial Design | 2030-2032 | 1. Implement a biomarker-driven master platform trial allowing biomarker-driven arm assignment based on ecosystem state (immune-permissive vs. desert).  2. Retrospectively train computational digital twin models on phase I/II trial data. Validate predictions of response/resistance in phase II adaptive expansion cohorts.  3. Submit spatial biomarkers for FDA qualification as exploratory endpoints. |

*Abbreviations: mCRPC, metastatic castration-resistant prostate cancer; ctDNA, circulating tumor DNA; IL-6, interleukin-6; TGF-β, transforming growth factor-beta; CAF, cancer-associated fibroblast; ARPI, androgen receptor pathway inhibitor; iCAF, inflammatory CAF; mCAF, myofibroblastic CAF; PSMA, prostate-specific membrane antigen; TIL, tumor-infiltrating lymphocyte; FAP, fibroblast activation protein; CAR T, chimeric antigen receptor T cell; IND, Investigational New Drug; FDA, U.S. Food and Drug Administration.*
